# Application of decision tree in determining the importance of surface electrohysterography signal characteristics for recognizing uterine contractions

**DOI:** 10.1016/j.bbe.2019.06.008

**Published:** 2019

**Authors:** Dongmei Hao, Qian Qiu, Xiya Zhou, Yang An, Jin Peng, Lin Yang, Dingchang Zheng

**Affiliations:** a College of Life Science and Bioengineering, Beijing University of Technology, Intelligent Physiological Measurement and Clinical Translation, Beijing International Base for Scientific and Technological Cooperation, Beijing, China; b Department of Obstetrics, Peking Union Medical College Hospital, Beijing, China; c Health and Wellbeing Academy, Faculty of Medical Science, Anglia Ruskin University, Chelmsford, United Kingdom

**Keywords:** Electrohysterogram (EHG), Decision tree, Uterine contraction, Importance

## Abstract

The aims of this study were to apply decision tree to classify uterine activities (contractions and non-contractions) using the waveform characteristics derived from different channels of electrohysterogram (EHG) signals and then rank the importance of these characteristics. Both the tocodynamometer (TOCO) and 8-channel EHG signals were simultaneously recorded from 34 healthy pregnant women within 24 h before delivery. After preprocessing of EHG signals, EHG segments corresponding to the uterine contractions and non-contractions were manually extracted from both original and normalized EHG signals according to the TOCO signals and the human marks. 24 waveform characteristics of the EHG segments were derived separately from each channel to train the decision tree and classify the uterine activities. The results showed the Power and sample entropy (SamEn) extracted from the un-normalized EHG segments played the most important roles in recognizing uterine activities. In addition, the EHG signal characteristics from channel 1 produced better classification results (AUC = 0.75, Sensitivity = 0.84, Specificity = 0.78, Accuracy = 0.81) than the others. In conclusion, decision tree could be used to classify the uterine activities, and the Power and SamEn of un-normalized EHG segments were the most important characteristics in uterine contraction classification.

## 1 Introduction

Uterine contraction provides physiological information of the uterine activity and plays an important role in monitoring the health of mother and fetus during pregnancy. It also helps clinicians to distinguish the normal contraction from the contraction that may lead to preterm labor. The process of labor and delivery can be monitored by regular uterine contraction and cervical dilation [1]. At the preparatory phase, uterine contraction evolves from an inactive to active state [2], [3]. When the delivery approaches, uterine contraction becomes more and more synchronized, which finally leads to the expulsion of fetus [4].

Tocodynamometer (TOCO) and internal uterine pressure (IUP) catheter are conventionally used to monitor the uterine contraction during pregnancy and labor. However, the TOCO measurement largely depends on the subjective experience of the examiners as well as the sensor position [3]. Since TOCO mainly provides the frequency information of uterine contractions, it is therefore often used as a counter of uterine contractions and fails to provide sufficient clinical information to differentiate the abnormal uterine activities from the normal ones. In terms of IUP measurement, it not only monitors the IUP changes during uterine activities [5], [6], but also provides reliable information about the uterine dynamics [7]. However, the rupture of membranes is required during IUP measurement. Therefore, it cannot be routinely used to monitor the uterine contraction.

Published studies have shown that electrohysterogram (EHG) signals recorded noninvasively from the maternal abdomen can provide information about electrical activity of myometrial cells that leads to uterine contractions [8]. EHG measurement has been considered as an alternative of TOCO and an effective tool to monitor the uterine contractions [3], [9], [10], [11]. It has been reported that EHG signal characteristics (e.g. the root mean square (RMS) value) in association with uterine contraction had high correlation with the outcome from the TOCO measurement [12]. Previous studies have also demonstrated that various EHG signal characteristics (e.g. RMS, median frequency, peak frequency, log detector, etc.) and their combinations could be used to recognize contractions during pregnancy [13], [14], [15] and detect preterm labor [16], [17], [18]. However, there were few studies with the primary aim to distinguish contraction signals and non-contraction signals using the characteristics extracted from the EHG.

Previous studies have mainly employed neural network to detect preterm/term labor [16], [19], [20] or support vector machine to discriminate uterine contractions between pregnancy and labor [14], [21] and detect preterm labor [13]. The decision tree is another technique for predicting and mining data which can be applied in classification, clustering and prediction tasks, especially in classification problems. It has been successfully applied on electrocardiograph (ECG) signals to detect myocardial infarction [22] and arrhythmia [23], but has not been widely used in EHG recognition. Only one study was found, where the decision tree was used to distinguish preterm and term labor [24]. More importantly, with the application of decision tree, the characteristics importance could be ranked, and then the important characteristics could be selected.

The aims of this study were to apply decision tree to classify uterine activities (contractions and non-contractions) using the waveform characteristics derived from different channels of EHG signals and then rank the characteristics importance.

## 2 Materials and methods

### 2.1 EHG recording

8-Channel EHG signals were recorded from 34 healthy pregnant women (aged 30 ± 4 years old) within 24 h before delivery at Beijing Union Medical College Hospital in China. Their gestation age ranged between 38 and 41 weeks. All the pregnant women had no history of diseases, such as diabetes, hypertension and other known diseases in their medical records. The study was approved by the Local Ethics Committee of Beijing Union Medical College Hospital, and was conducted strictly according to the Declaration of Helsinki (1989) of the World Medical Association. The pregnant women were asked to sign consent after being informed of the aims, potential benefits and risks of the study.

**Fig. 1:**
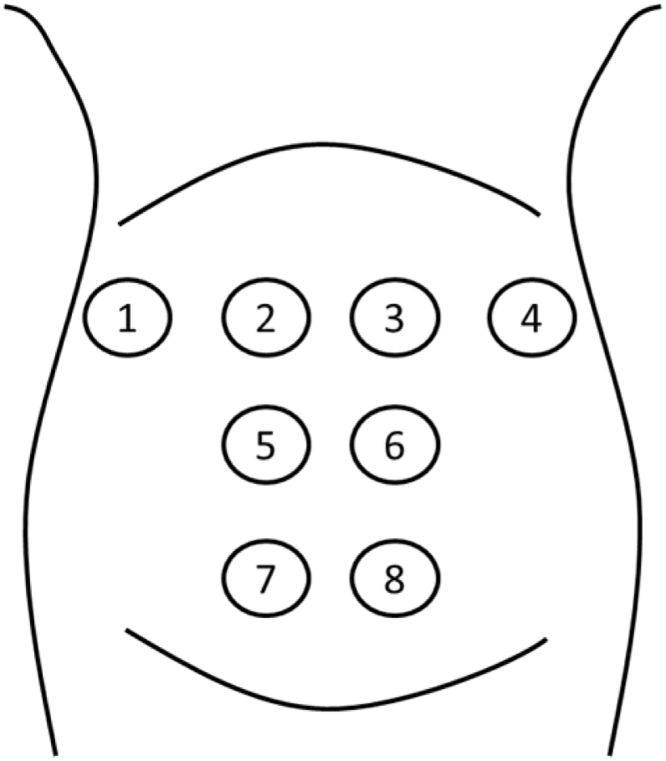
The arrangement of the eight electrodes on the abdomen.

TOCO and 8-channel EHG signals were recorded by the device designed in our lab. The technical parameters of this device were as follows: bandwidth of the EHG channels is 0–70 Hz, gain of amplifiers is 24, sampling rate is 250 Hz. And analog-to-digital converter (ADC) applied in this device is ADS1299 which is a 24-bit delta-sigma ADC. The uterine contractions were marked by the pregnant women when the uterine contractions were felt during signal recording. The arrangement of the eight electrodes is shown in Fig. 1. Electrode 1 and electrode 4 were placed on the abdomen wall upon the right and left uterine horns, respectively. Electrode 2 and electrode 3 were evenly placed along the fundus of the uterus. Electrode 7 and electrode 8 were placed on the abdomen wall upon the cervix, and electrode 5 and electrode 6 were evenly placed upon the uterine body between the fundus and cervix. This arrangement ensured that the whole uterus was covered by these electrodes. The reference and ground electrodes were placed upon the right and left ilium of the subject respectively.

### 2.2 EHG signal preprocessing and segmentation

The recorded EHG signals were firstly preprocessed by a low-pass filter (0–3 Hz) and a median filter to remove the unwanted signals, including the baseline drift, power line interference and maternal electrocardiogram (mECG). It was then normalized in amplitude between −1 and 1, separately for each of the 8-channel EHG signals. The reason for normalizing EHG signal was that, for the same waveform characteristic being derived from the EHG signals at the later processing step, different values would be obtained from normalized or un-normalized EHG signals.

**Fig. 2:**
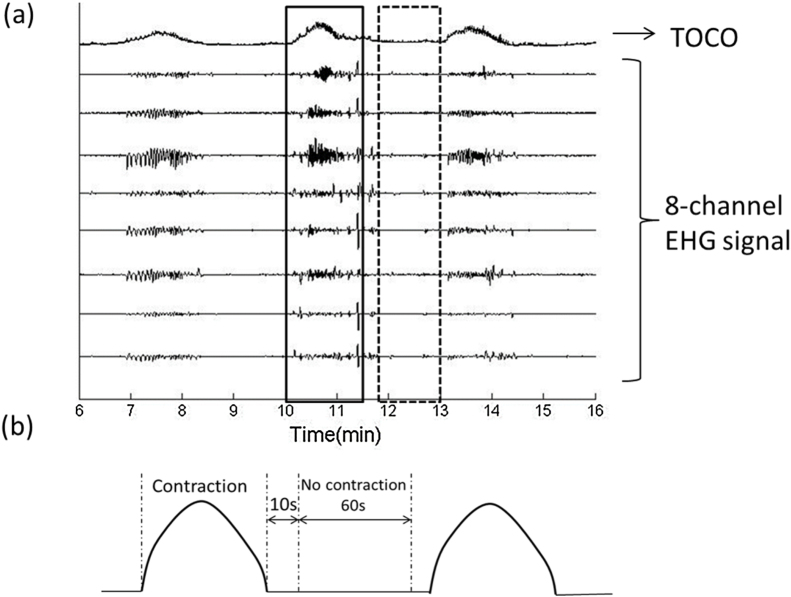
(a) Example waveforms of recorded TOCO signal; 8-channel EHG signals, and (b) the timing reference in the TOCO signal used to segment contraction and non-contraction EHG.

Next, the uterine contractions were manually determined and agreed according to the TOCO signal and the marks made by pregnant women. Fig. 2(a) shows an example of the recorded TOCO signal and 8-channel EHG signals from one subject. The EHG segmentation corresponding to the uterine contractions was then extracted from both the un-normalized and normalized EHG signals. They were simplified below as the ‘un-normalized and normalized EHG contraction segments’. The duration of the contraction is about 30–60 s clinically. And the stationary non-contraction period could be obtained 10 s after contraction. The duration of non-contraction was selected as 60 s to match the contraction duration. As a result, corresponding non-contraction period (60 s) was then extracted 10 s after the end of that contraction as shown in Fig. 2(b). In total, from each channel, 136 segments of contraction signals and 136 segments of non-contraction signals were obtained from normalized and un-normalized EHG signals, respectively. The number of all EHG segments ranged from 1 to 8 for each individual.

### 2.3 Waveform characteristics derived from each EHG segment

14 waveform characteristics were chosen to train the decision tree, including RMS, Standard Deviation (STD), log detector (LOG), mean absolute value (MAV), simple square integral (SI), difference absolute standard deviation value (DAS), average amplitude change (AAC), variance (VAR), median frequency (MF), peak frequency (PF), Power, time reversibility (TR), Lyapunov exponents(Ly)and sample entropy (SamEn) [13], [16], [25], [26]. These characteristics were calculated as follows:

(1) $$\text{RMS}=\sqrt{\frac{1}{N}\sum_{i=1}^{N}x{\left(i\right)}^{2}}$$

(2) $$\text{STD}=\sqrt{\frac{1}{N}\sum_{i=1}^{N}\left(x(i)-\frac{1}{N}\sum_{i=1}^{N}x(i)\right)}$$

(3) $$\text{LOG}=e^{1/N\sum_{i=1}^{N}\log(|x(i)|}$$

(4) $$\text{MAV}=\frac{1}{N}\sum_{i=1}^{N}\left|x(i)\right|$$

(5) $$\text{SI}=\sum_{i=1}^{N-1}{\left|x(i)\right|}^{2}$$

(6) $$\text{DAS}=\frac{1}{N-1}\sum_{i=1}^{N-1}{\left(x(i+1)-x(i)\right)}^{2}$$

(7) $$\text{AAC}=\frac{1}{N}\sum_{i=1}^{N-1}\left|x(i+1)-x(i)\right|$$

(8) $$\text{VAR}=\frac{\sum_{i=1}^{N}{\left(x(i)-\mu \right)}^{2}}{N}$$

(9) $$\text{PF}=\arg\left(\frac{f_{s}}{N}\overset{N}{\underset{i=1}{\max}}P(i)\right)$$

(10) $$\text{MF}=i_{m}\frac{f_{s}}{N}\sum_{i=1}^{i=i_{m}}P(i)=\sum_{i=i_{m}}^{i=N}P(i)$$

(11) $$\text{Power}=\sum_{i=1}^{N}P(i)/N/f_{s}$$

(12) $$\text{TR}=\left(\frac{1}{N-\tau }\right)\sum_{i=\tau +1}^{N}{\left(x(i)-x(i-\tau )\right)}^{3}$$

(13) $$\text{LE}=\underset{t\to \infty }{\lim}\underset{∥\Delta x(t_{0})∥\to 0}{\lim}\left(\frac{1}{t}\right)\log(∥\Delta x(t)∥/∥\Delta x(t_{0})∥)$$

where *x*(*i*) is the segmented EHG, *i* = 1, 2, …, *N.* *N* is the length of the *x*(*i*). $\mu =\sum_{i=1}^{N}x(i)/N$ is the mean of the *x*(*i*). *P*(*i*) represents the power spectrum of *x*(*i*). *f*_s_ is the sampling frequency. τ represents the time delay. $∥\Delta x(t_{0})∥$ and $∥\Delta x(t)∥$ are the Euclidean distance between two states of *x*(*i*), respectively to an arbitrary time *t*_0_ and a later time *t*. In this study *τ* = 1.

When it comes to SamEn, template vectors are defined as *y*(*m*) = (*x*_*i*_, *x*_*i*+1_, …, *x*_*i*+*m*−1_), *i* = 1, 2, …, *N* − *m* + 1, *m* is the length of the template.

(14) $$B^{m}\left(r\right)=\frac{1}{N-m+1}\sum_{i=1}^{N-m+1}B_{i}^{m}\left(r\right)$$

where $B_{i}^{m}\left(r\right)$ is the number of vectors *y*(*j*) within r of *y*(*i*), *j* ranges from 1 to N-m, and i≠j. Then the length of the template was defined as *m* + 1, the process describe above was repeated, and $B^{m+1}\left(r\right)$ was obtained.

(15) $$SamEn(m,r,N)=-\ln\left[B^{m+1}\left(r\right)/B^{m}\left(r\right)\right]$$

In this study, *m* = 8, *N* = 500, and r is the location where the first zero-crossing point in the correlation of the two templates is.

All these 14 waveform characteristics were extracted from both normalized and non-normalized EHG segments except for Ly. Ly was only calculated on the un-normalized EHG segments because that the normalization process changed the autocorrelation coefficient of the EHG signals and made Ly inaccurate. Furthermore, the values of MF, PF and SamEn of normalized EHG segments were the same as those extracted from un-normalized EHG segments. Therefore, the total number of characteristics used for classification was 24.

### 2.4 Classification of uterine contraction

It is known that the classification results based on the decision tree are influenced by the number and type of attributes. In this study, the classification and regression tree (CART) was trained and tested using 24 waveform characteristics derived from the EHG segments. This approach was performed separately on EHG segments from each of the 8 channels. 10-Fold cross validation was employed with all the 272 segments divided into 10 groups. One group was selected to perform as the testing data, and the remaining 9 groups were used as the training data each time.

During the decision making process, different EHG signal characteristics had different degrees of influence on the decision, which was quantified by the importance index extracted from the decision tree, allowing the characteristics importance to be ranked. As a result, the most important and dominant characteristics were selected.

The decision tree was normally pruned at the best level to achieve the best classification outcome. The averaged best level was then calculated from the 10-fold cross validation, separately for each channel.

### 2.5 Classification evaluation

The indices for evaluating the performance of the classifier include the area under receiver operating characteristic curve (AUC), sensitivity, specificity, positive predictive value (PPV), negative predictive value (NPV) and accuracy. Sensitivity, specificity, PPV, NPV and accuracy were calculated as below:

(16) sensitivity=TPR=TP/(TP+FN)

(17) specificity=1−FPR=TN/(TN+FP)

(18) PPV=TP/(TP+FP)

(19) NPV=TN/(TN+FN)

(20) Accuracy=(TP+TN)/(TP+TN+FP+FN)

where TP (true positive) and TN (true negative) are the numbers of contraction and non-contraction EHG segments respectively that were correctly classified, FP (false positive) and FN (false negative) are the numbers of contraction and non-contraction EHG segments respectively that were falsely classified. As a result of the 10-fold cross validation, 10 values of AUC, sensitivity, specificity, PPV, NPV and accuracy values were obtained, separately for each channel of the EHG signals. Kruskal–Wallis test was employed using software SPSS 23 (SPSS Inc.) to assess the repeatability between the results of the 10-fold cross validation. The average of the 10 values was then calculated to evaluate the classification results, respectively for each channel.

## 3 Results

### 3.1 Characteristics importance in the decision tree

**Fig. 3:**
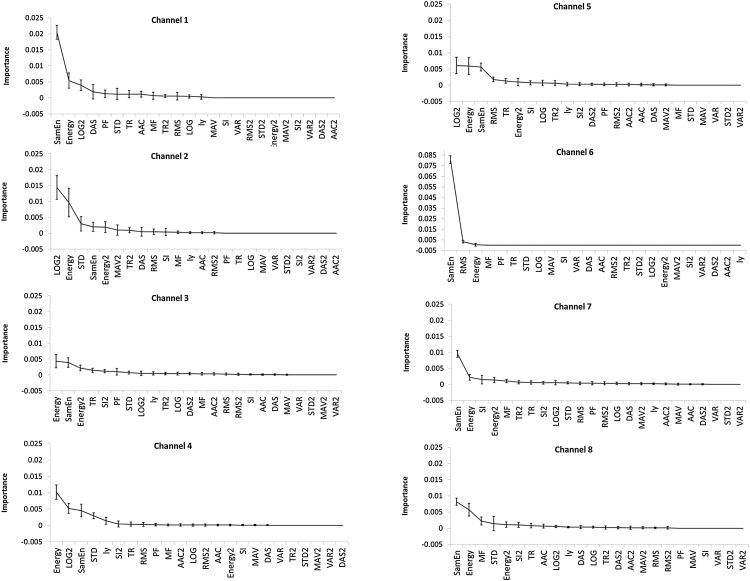
Importance of different characteristics in decision tree. The data is shown as mean ± SD. RMS, MF, PF, LOG, SI, MAV, DAS, AAC, STD, VAR, SamEn, TR, Power and Ly were extracted from the un-normalized EHG segments. RMS2, LOG2, SI2, MAV2, DAS2, AAC2, STD2, VAR2, TR2 and Power2 were extracted from the normalized EHG segments. SD was calculated from the 10-fold cross validation.

During the process of pruning, the averaged best levels were 2.1, 1.3, 3.3, 2.5, 2.2, 0.9, 3.4, 2.0 for Channel 1 to Channel 8, respectively. The importance of EHG signal characteristics for classifying uterine contraction and non-contraction was separately plotted for each channel of EHG, as shown in Fig. 3. It is shown that not all the characteristics of EHG segments played similar important roles in classification, and the importance of each characteristic was different for each channel. The top 4 important characteristics for each channel are summarized in the Table 1. The Power and sample entropy (SamEn) extracted from the un-normalized EHG segments were two of the most important characteristics in recognizing uterine contractions and non-contractions. This was observed from every channel of the EHG signals.

**Table 1:** Summary of the top 4 important characteristics of EHG segments of each channel.

Channel	Power	SamEn	LOG2	STD	Power2	RMS	MF	TR	SI	DAS
Ch1	√	√	√							√
Ch2	√	√	√	√						
Ch3	√	√			√			√		
Ch4	√	√	√	√						
Ch5	√	√	√			√				
Ch6	√	√				√	√			
Ch7	√	√			√				√	
Ch8	√	√		√			√			

### 3.2 Classification results

There were no significant differences in the classification results (AUC, Sensitivity, specificity, PPV, NPV) between the ten values from the 10-fold cross validations (all *p* > 0.05), demonstrating the reliability of the decision tree. Table 2 illustrates their mean values obtained from the 10-fold cross-validation. It can be seen that Channel 6 produced the highest sensitivity and NPV. However, it performed poorly in specificity and PPV. Overall, channel 1 had relatively better classification performance in the decision tree (AUC = 0.75, Sensitivity = 0.84, Specificity = 0.78, PPV = 0.80, NPV = 0.83, Accuracy = 0.81).

**Table 2:** Classification performance of the decision tree for classifying uterine activities, separately for each of the 8 channels EHG signals.

Channel	AUC	Sensitivity	Specificity	PPV	NPV	Accuracy
Ch1	0.75	0.84	0.78	0.80	0.83	0.81
Ch2	0.67	0.70	0.72	0.73	0.72	0.71
Ch3	0.65	0.68	0.74	0.74	0.70	0.71
Ch4	0.62	0.66	0.66	0.66	0.67	0.66
Ch5	0.70	0.74	0.76	0.76	0.75	0.75
Ch6	0.69	0.97	0.53	0.68	0.95	0.75
Ch7	0.67	0.65	0.78	0.76	0.69	0.72
Ch8	0.69	0.68	0.76	0.76	0.71	0.72

## 4 Discussion

In this study, decision tree has been applied to classify the uterine contraction and non-contraction segments using 24 EHG characteristics. The classification results differed between different channels of EHG signals, and the Power and SamEn of un-normalized EHG segments played the most important roles in recognizing the contraction and non-contraction EHG segments.

Several methods have been proposed to detect the uterine contraction through the reconstruction of IUP-like or TOCO-like signals. These IUP-like or TOCO-like signals can be estimated by calculating RMS of EHG signals in sliding window [27], using FIR Wiener filter [28], or un-normalized first statistical moment of the frequency spectrum [29]. There were good correlations between the reconstructed IUP-like signal and the reference IUP measured by the IUP catheter. The key difference between the earlier studies and this work is that the proposed method in this work was based on the analysis of waveform characteristics of EHG segments instead of the reconstructed TOCO-like or IUP-like signals. Previous study obtained Accuracy = 87% in recognizing uterine contraction by calculating RMS to construct TOCO-like signals [27]. In [28] and [29], the uterine contraction was recognized by the reconstructed IUP-like signals. However the method proposed in this paper distinguished contraction signals and non-contraction signals using the characteristics extracted from the EHG. In our paper, the best performance of the decision tree is AUC = 0.75, Sensitivity = 0.84, Specificity = 0.78, PPV = 0.80, NPV = 0.83, Accuracy = 0.81. Compared to Accuracy = 87% in [27], the Accuracy in our study is lower. This is partly because that [27] only considered contraction signals, however we took the non-contraction signals in consideration. The signals which were misclassified came from not only contraction signals but also non-contraction ones.

It has been studied that the quality of EHG signals and the accuracy of classifying contractions during pregnant and labor varied with the position of EHG electrodes. The uterine median axis and the lower center-right umbilical region have been demonstrated to be the optimal position for recording EHG signal [7]. But other studies reported that the channels located near the median axis of the uterus provided poorer result in classifying uterine contraction than those at the extremities [14]. Our study partially agreed with the results described in [14]. The electrode at the right uterine horn (channel 1) in this study provided better result in classifying the contraction and non-contraction activities. The inconsistent results of which position is better for EHG recording or for classifying uterine contractions could be explained by the different EHG signals. The EHG signals used in [7] were recorded during 37–41 weeks. And the EHG signals used in [14] were recorded during pregnancy and labor. However in this study, the EHG signals were recorded from the pregnant women within 24 h before delivery.

More importantly, this study ranked the characteristics importance in the decision making process. This would allow further studies to be more specific in selecting characteristics and reduce the number of characteristics for training a classifier to recognize contraction signals. In this way, the classifier with simple structure will be achieved, and less time is required on training and testing. It has been shown in this study that the Power and SamEn extracted from un-normalized EHG segments played crucial roles for all channels. Previous studies reported that, when the delivery is approaching, the Power [9], [15] and SamEn [24] of EHG signals changed significantly with increased frequency of uterine contractions. This may implicate that the two waveform characteristics should be more significantly different between contraction and non-contraction.

**Fig. 4:**
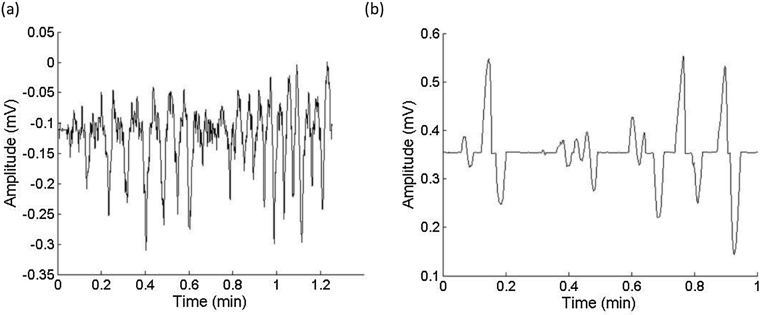
Examples of EHG segments that were classified falsely: (a) contraction signal that was falsely classified; (b) non-contraction signal that was falsely classified.

The present work has some limitations. Firstly, the EHG signal characteristics employed in this study were commonly used to predict preterm birth [16]. It is not sure whether they are the best ones for classifying the uterine contraction and non-contraction activities. Other waveform characteristics could be explored and added in the future. Secondly, it is noticed that some EHG segments contained noise. Fig. 4 shows a couple of examples of the contraction and non-contraction EHG segments that were falsely classified. The source of the noise in the EHG signal has not been fully understood. New methods need to be developed in the future studies to remove their effect on classification.

## 5 Conclusions

In conclusion, this study has demonstrated that the decision tree could be used to classify the contraction and non-contraction activities from EHG signals, and the Power and SamEn of un-normalized EHG segments have been shown to be the most important characteristics in the uterine contraction classification.

## Data availability

The datasets generated and analyzed during the current study are not publicly available because that the data is unique to our study but are available from the corresponding author on reasonable request.

## Funding statement

This research was sponsored by Bill & Melinda Gates Foundation [OPP1148910] and Beijing Natural Science Foundation [7172015].

## Ethical guidelines

All procedures performed in studies involving human participants were in accordance with the ethical standards of local ethics committee of Beijing Union Medical College Hospital and with the Declaration of Helsinki (1989) and its later amendments or comparable ethical standards. This article does not contain any studies with animals performed by any of the authors.

## Author's contribution

The contributions of every author are as follows:

Dongmei Hao: conceptualization, writing – review & editing

Qian Qiu: methodology, software, writing – original draft

Xiya Zhou: investigation, resources

Yang An: data curation

Jin Peng: validation

Lin Yang: data curation

Dingchang Zheng: conceptualization, writing – review & editing
